# Impact of Ischemia-Reperfusion on Extracellular Matrix Processing and Structure of the Basement Membrane of the Heart

**DOI:** 10.1371/journal.pone.0092833

**Published:** 2014-03-28

**Authors:** Alexander Lauten, Alexandra Gerhard-Garcia, Frank Suhr, Juergen H. Fischer, Hans R. Figulla, Wilhelm Bloch

**Affiliations:** 1 Department of Internal Medicine I (Cardiology, Angiology, Pneumology), Friedrich- Schiller University, Jena, Germany; 2 Institute of Cardiovascular Research and Sports Medicine, German Sports University, Cologne, Germany; 3 Institute for Experimental Medicine, University of Cologne, Cologne, Germany; KRH Robert Koch Klinikum Gehrden, Germany

## Abstract

**Purpose:**

Acute ischemic injury is a strong inductor of cardiac remodelling, resulting in structural changes of the extracellular matrix (ECM) and basement membrane (BM). In a large animal model of ischemia-reperfusion (I/R) we investigated the post-ischemic liberation of the collagen-IV-fragments Tumstatin (TUM; 28 kDa-fragment of collagen-IV-alpha-3), Arresten (ARR; 26 kDa-fragment of collagen-IV-alpha-1) and Endorepellin (LG3, 85 kDa-fragment of perlecan) which are biologically active in angiogenesis and vascularization in the post-ischemic myocardium.

**Methods and Results:**

In this blinded study, 30 pigs were randomized to 60 min of global I/R at either 4°C or 32°C or served as control. Three transmyocardial tissue samples were collected prior to ischemia and within 30 min and 150 min of reperfusion. Tissue content of TUM, ARR and LG3 was analyzed by western blotting and immunostaining.

Within 150 min of mild hypothermic I/R a significantly increased tissue content of ARR (0.17±0.14 vs. 0.56±0.56; p = 0.001) and LG3 (1.13±0.34 vs. 2.51±1.71, p<0.001) was observed. In contrast, deep hypothermic I/R was not associated with a significant release of cleavage products. Cleavage of TUM remained unchanged irrespective of temperature. Increased matrix processing following mild hypothermia I/R is further supported by a >11fold elevation of creatine kinase (2075±2595 U/l vs. 23248±6551 U/l; p<0.001) in the coronary sinus plasma samples. Immunostaining demonstrated no changes for ARR and LG3 presentation irrespective of temperature. In contrast, TUM significantly decreased in the BM surrounding cardiomyocytes and capillaries after mild and deep hypothermic I/R, thus representing structural alterations of the BM in these groups.

**Conclusion:**

The study demonstrates an early temperature-dependent processing of Col-IV as major component of the BM of cardiomyocytes and vascular endothelium. These observations support the protective effects of deep hypothermia during I/R. Furthermore, the results suggest an increased structural remodelling of the myocardial basement membrane with potential functional impairment during mild hypothermic I/R which may contribute to the progression to post-ischemic heart failure.

## Introduction

Collagen IV (Col-IV) and perlecan (Hspg2) are major structural components of the basement membrane (BM) surrounding cells and cell layers in the myocardium. By interacting with other components of the extracellular matrix (ECM), Col-IV and Hspg2 modulate cardiomyocyte activity and are essential for normal tissue development and function. Both proteins act by linking myocytes to the surrounding ECM and interstitial cells, thus transmitting mechanical force during contraction [3], [4]. Both proteins are therefore essential for mechanical stability and electrical conduction in the myocardium [15], [23], [25]. Conditions leading to proteolysis of Col-IV and Hspg2 may potentially result in functional impairment of the myocardium.

Myocardial ischemia-reperfusion (I/R) is a strong inductor of ECM remodelling by initiating a series of events leading to protein cleavage and re-formation of the ECM [12]. The cleavage of Col-IV liberates the C-terminal fragments arresten (ARR, collagen-IVα1) and tumstatin (TUM, collagen-IVα3), while proteolysis of Hspg2 results in liberation of the C-terminal domain endorepellin (LG3). Multiple biological activities such as the suppression of angiogenesis by inhibiting endothelial cell migration, tube formation and pro-apoptotic function have been attributed to these fragments, [2], [6], [10], [14], [22], [28]. However, the role of ARR, TUM and LG3 in myocardial vascularization following acute ischemia has never been studied.

The purpose of this study is to investigate the time-dependent cleavage of Col-IV and Hspg2 during early I/R with release of the above bioactive fragments under different temperature conditions. We hypothesize a differential cleavage of Col-IV and Hspg2 during mild and deep hypothermic I/R as potential protective strategy [2], [16], [17], [19].

## Materials and Methods

### 2.1 Anesthesia and Operative Procedure

Thirty female pigs (23±3 kg) were included in this study. All animal work has been conducted according to relevant national and international guidelines. All animals were housed and fed according to the National Institutes of Health Guide for the Care and Use of Laboratory Animals (NIH Publication 85-23, revised 1985) federal guidelines. All procedures were approved by the Ethics Committee of the Animal Welfare Authorities Thuringia (TLLV) [12]. In brief, in all animals anesthesia was induced with 4 mg/kg intramuscular azaperon (Stresnil, Janssen, Neuss, Germany) and 0.02 mg/kg atropine (Braun, Melsungen, Germany) and maintained with 0.03–0.05 mg/kg/min ketamine (Ketanest, Parke-Davis, Berlin, Germany). Pancuronium 0.2 mg/kg was given as required. The animals were mechanically ventilated with 50% oxygen in room air. For hemodynamic monitoring, fluid administration and blood sampling fluid-filled catheters were inserted in the right common carotid artery and the right internal jugular vein, respectively. Following left anterior thoracotomy in the fourth intercostal space and pericardiotomy, heparin (300 IU/kg) was given for systemic anticoagulation. The extracorporeal circuit and the membrane oxygenator (Cobe Cardiovascular Inc., Arvada, CO, USA) were primed with heparinized pig blood. Non-pulsatile cardiopulmonary bypass (CPB) was initiated at a flow rate of 100 ml/kg/min and adjusted to maintain a minimum mean arterial pressure of 50 mmHg. After start of CPB animals were randomly assigned to one of three study groups.

### 2.2 Study design

Animals in group one (n = 9) served as controls. They underwent 60 min of CPB without myocardial ischemia. In 21 animals, myocardial ischemia was induced by aortic cross clamping and infusion of potassium solution into the aortic root until electrical and mechanical arrest of the heart. Depending on protocol, either deep hypothermic (group DeepH; approx. 4°C) or mild hypothermic (group MildH; approx 32°C) conditions were then sustained during myocardial ischemia. In group two myocardial protection was additionally afforded by topical cooling, maintaining a solution of iced saline in the pericardium during the 60 min interval. After 60 min of ischemia, the cross-clamp was removed and the heart was reperfused. The animals were weaned off CPB and all cannulas were removed.

Transmural myocardial biopsies were collected from a fat-free area of the mid-left ventricular free-wall at the following time points: at baseline prior to 60 min of global myocardial ischemia and following ischemia at 30 min and 150 min of reperfusion. At each time point, two LV biopsies were snap frozen in liquid nitrogen and stored at −80°C or fixed in 4% paraformaldehyde. At 30 min and 150 min of reperfusion venous blood samples were collected from the coronary circulation in a vacutainer system for further analysis. After conclusion of all measurements and sampling animals were euthanized with anesthesia overdose and intravenous potassium.

### 2.3 Western Blot analysis

Proteins of tissue homogenates were separated on SDS-PAGE (Bio-Rad, Copenhagen, Denmark). After transfer with a semi-dry transblot apparatus (Bio-Rad, Copenhagen, Denmark) the membranes were blocked with 1% nonfat dry milk for one hour at room temperature. The membranes were each incubated with the specific primary antibody (Millipore, Schwalbach, Germany) at 4°C overnight followed by washing three times in wash buffer I for 15 min at room temperature. They were then incubated in goat anti-rabbit secondary antibody (DAKO, Hamburg, Germany). After washing with horseradish peroxidase complex (Amersham) for one hour, each protein was visualized using DAB solution [1%DAB and 0.75% H2O2 in 0.1M PB (pH 7.4)]. Protein content was quantified by densitometric measurement. Actin was used as loading control.

### 2.4 Measurement of Creatine kinase Serum Levels

Serum levels of creatine kinase (CK in U/l) were measured by an enzymatic assay (Bayer Diagnostics, Leverkusen, Germany) in venous blood samples from the coronary circulation at 30 min and 150 min of reperfusion. Nine and a half milliliters of blood was collected in a vacutainer system (Becton Dickinson), centrifuged for 10 min at 1.861*g and 4°C and stored at −80°C until further analysis.

### 2.5. Immunohistochemistry (IHC) for Collagen IV- and Hspg2 Fragments

After fixation in 4% paraformaledehyde tissue specimens were extensively washed in 0.1M PBS pH 7.4, dehydrated in grading alcohol series including a 50%, 70%, 90% and 100% ethanol step, rinsed in chloroform, embedded in paraffin and cut in 5 µm sections on a conventional microtome. Sections were incubated with 5% bovine serum albumin (Sigma, Germany) in TBS for one hour at RT and then incubated with 1∶600 rabbit antibodies against ARR, TUM, LG3, TIMP IV and cathepsin. After rinsing with PBS, slides were incubated for 1 h in a 1∶400 dilution of a biotinylated anti-rabbit antibody (Dako, Glostrup, Denmark) and a horseradish-peroxidase complex (1∶150 diluted) then used as detection system (Amersham, England). The reaction products were visualized with 3.3-diaminobenzidine-tetrahydrochloride in 0.05M Tris-HCL buffer containing 0.1% hydrogen peroxide. For staining intensity detection, a Leica microscope coupled to a CCD-camera (DXC-1850P, Sony, Germany) was used and the analysis was conducted using the software “Image J” (National Institutes of Health, Bethesda, Maryland, USA). The background gray value was detected at a cell-free area of the slide and was equalized for each slide prior to image capturing. Differences in staining intensity between time points were determined as the difference between background intensity and test area. Digitally captured images (63× magnification) were analyzed to determine cleavage fragment densities in BM of endothelial cells. In all experiments staining without the primary antibody was used as a negative control.

### 2.6 Statistical analysis

Statistical analysis was performed by using a statistics software package (SPSS for Windows, Version 15.0). All data are presented as mean±SEM. Data analysis was performed using two-*way ANOVA* with Bonferroni post hoc test or student's *t-test* for paired data as appropriate. Statistical differences were considered to be significant for values of p<.05.

## Results

### 3.1 Changes of Hemodynamics and Temperature throughout the Experiment

No significant differences in systemic hemodynamics between groups were observed throughout the experiment. Basic hemodynamic parameters and temperature changes are given for representative time points in table 1. In group three (normothermia), five animals were only partially weaned from CPB due to myocardial failure; in these animals tissue samples were collected during reperfusion with continuous extracorporal circulatory support. Myocardial temperature was monitored during ischemia by temperature probes placed in the anterior LV free wall. According to the study protocol, a progressive reduction of myocardial temperature to deep hypothermic conditions was achieved in DeepH by topical cooling. Between MildH and controls no significant difference in myocardial temperature was observed.

**Table 1:** Hemodynamics and Temperature Changes.

	Controls	Deep Hypothermia (4°C)	Mild Hypothermia (32°C)
**Baseline**			
MAP (mmHg)	72±11	74±9	69±13
HR (bpm)	108±10	103±7	110±8
Temperatur °C	37.1±1.4	37.6±1.4	36.2±2.0
***Lowest Temp. during CPB/ischemia***			
	33.7±2.5	10.1±3.0*	35.3±3.0
***30 min Reperfusion***			
MAP (mmHg)	*64±18*	*72±14*	*66±13*
HR (bpm)	*103±12*	*122±20*	*118±18*
Temperatur °C	34.6±2.4	29.0±3.1	36.6±2.7
***150 min Reperfusion***			
MAP (mmHg)	62±9	64±15	63±13
HR (bpm)	115±12	118±14	115±12
Temperatur °C	34.2±3.9	34.6±4.0	37.3±3.5

*Basic hemodynamic data and temperature for representative timepoints. No major differences of basic hemodynamics were observed between groups throughout the experiment.*

* Temperature significantly lower (p<.05 vs. control).

### 3.2 Serum Levels of Creatine kinase in the Coronary Sinus

**Figure 1:**
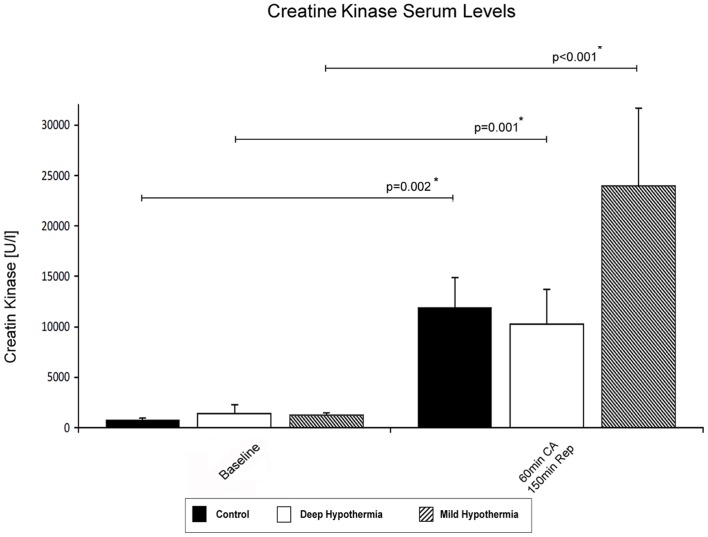
Serum levels of creatine kinase in samples collected at baseline and 150 min of reperfusion. A significant CK increase was found in all three groups. After mild hypothermic I/R a >11-fold CK increase was observed.

Serum levels of creatine kinase (CK) were determined as marker of myocardial injury in samples collected from the coronary sinus during reperfusion. In animals after mild hypothermic I/R a >11-fold increase in CK serum levels were noted at 150 min of reperfusion (2075±2595 U/l vs. 23248±6551 U/l; p<0.001). An increase of CK serum levels were also observed in the deep hypothermic group (1372±889 U/l vs. 10260±3416 U/l; p = 0.001) and in controls (1448±946 U/l vs. 11922±2949 U/l; p = 0.002), however, considerably less compared to mild hypothermic ischemia. The time course of CK serum levels is presented in figure 1.

### 3.3 Collagen IV- and Hspg2 Degradation during Ischemia-Reperfusion in Myocardial Tissue Samples

Western blot analysis was performed with myocardial tissue samples at baseline and at 30 min and 150 min of reperfusion to evaluate the release of the biologically active fragments of collagen IV and Hspg2.

#### Tissue Content of Arresten (collagen IV α1 chain)- Western Blot Analysis and IHC

**Figure 2:**
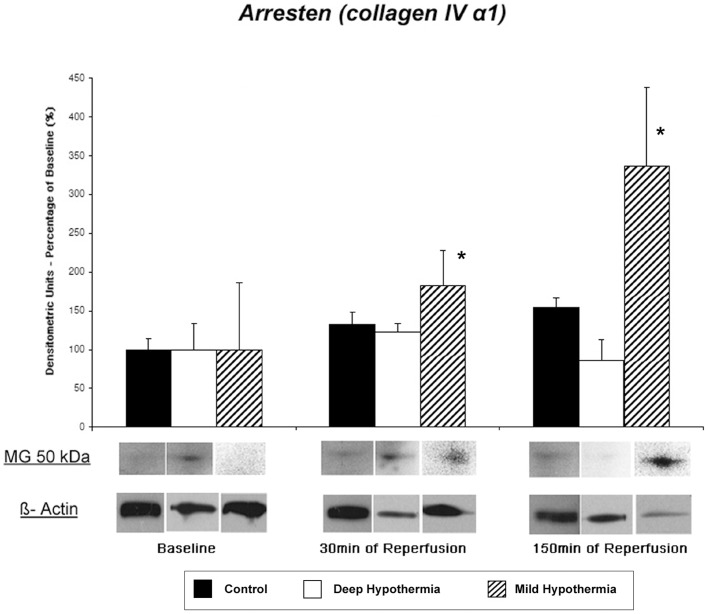
Myocardial tissue content of Arresten. Release patterns and representative western blot of the 50-fragment as strongest band identified by immunoblotting with arresten polyclonal antibody. * p = 0.001.

Arresten is the 26 kDa C-terminal (NC1) domain of the α1-chain of type IV collagen in the BM. Using a specific antibody, five bands were identified by their N-terminal sequence at 10 kDa, 20 kDa, 25 kDa, 50 kDa, and 75 kDa in all groups at baseline with strongest staining at 50 kDa (figure 2). At baseline a comparable protein content of ARR was found in all three groups at 50 kDa (Control: 0.17±0.02, DeepH: 0.16±0.05, MildH: 0.17±0.14 densitometric units, p = n.s.). During deep hypothermic I/R and in controls no significant difference in release of ARR was observed during reperfusion. In contrast, animals after mild hypothermic I/R demonstrated a significant increase of ARR tissue content [Control: 0.23±0.04 (30 min REP) vs. 0.27±0.03 (150 min REP, p = 0.072), DeepH: 0.19±0.02 (30 min REP) vs. 0.14±0.04 (150 min REP, p = 0.26), MildH: 0.30±0.13 (30 min REP) vs. 0.56±0.56 (150 min REP) densitometric units, p = 0.001)].

**Figure 3:**
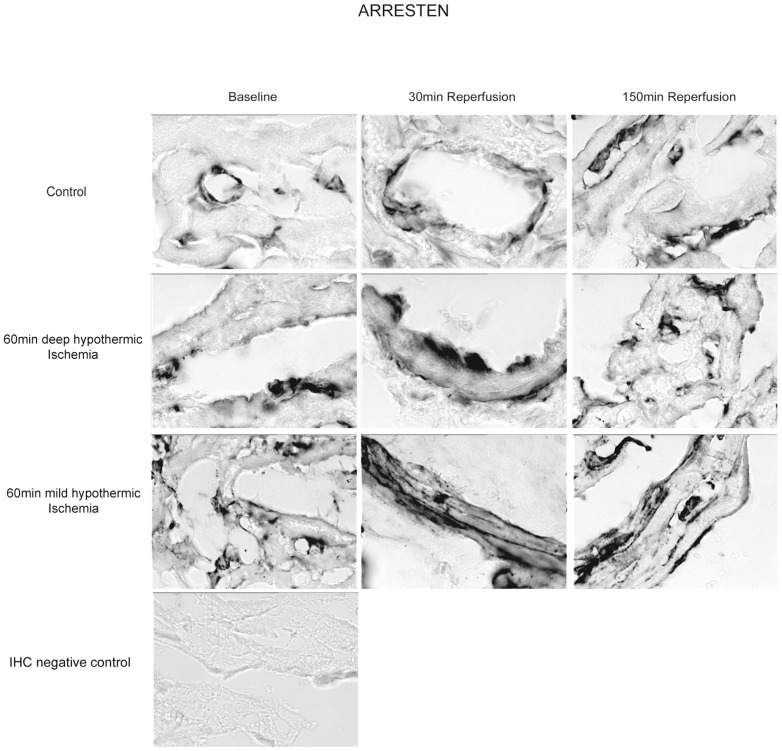
Immunohistochemical staining after deep hypothermic I/R for Arresten (Coll4α1) in myocardial sections at baseline, 30 min and 150 min of reperfusion. A significant decrease in antibody binding on the cellular BM is observed at 150/R, however not after deep hypothermic I/R and in controls.

IHC with a polyclonal antibody demonstrated staining localized at the endothelial basement membrane as histochemical sites of ARR (Figure 3). During deep hypothermic ischemia and in controls, no major change in ARR release was observed. In contrast, mild hypothermic ischemia resulted in a 1.7-fold increase of antibody binding for Arresten at the vascular and cellular BM (Table 2).

**Table 2:** Intensity of immunostaining for Endorepellin, Arresten and Tumstatin.

	Baseline	30 min Reperfusion	150 min Reperfusion	*P*	*p*
GROUP- *Fragment*				Base vs. 30 min Rep	Base vs. 150 min Rep
CONTROLS					
*Arresten*	1.00±0.20	1.09±0.16	1.26±0.04	n.s.	n.s.
*Tumstatin*	1.00±0.19	1.66±0.44	1.88±0.42	0.06	0.02
*Endorepellin*	1.00±0.62	1.26±0.84	0.94±0.62	n.s.	n.s.
DEEP HYPOTHERMIA 4°C					
*Arresten*	1.00±0.30	1.23±0.37	0.99±0.19	n.s.	n.s.
*Tumstatin*	1.00±0.23	0.76±0.37	0.442±0.17	n.s.	0.05
*Endorepellin*	1.00±0.21	1.84±1.34	1.111±0.78	n.s	n.s.
MILD HYPOTHERMIA (32°C)					
*Arresten*	1.00±0.92	1.42±0.37	1.70±0.86	n.s.	n.s.
*Tumstatin*	1.00±0.54	0.98±0.47	0.48±0.43	n.s.	0.06
*Endorepellin*	1.00±0.54	1.138±0.27	1.200±0.54	n.s.	n.s.

*Intensity of immunostaining for Endorepellin, Arresten and Tumstatin. IHC demonstrates a significant decrease of tumstatin antibody binding to >50% of baseline values during deep hypothermic and mild hypothermic I/R.*

#### Tissue Content of Tumstatin (Collagen IV α3) – Western Blot Analysis and IHC

**Figure 4:**
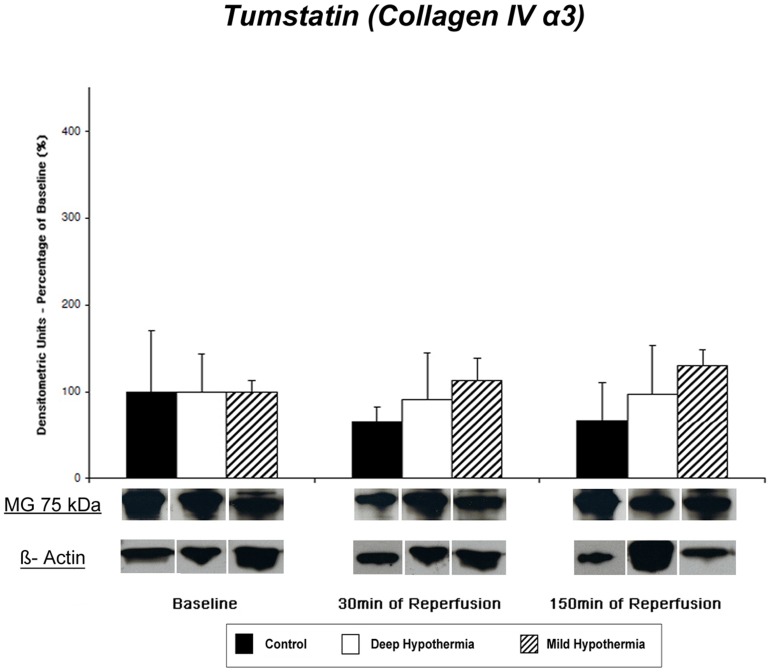
Myocardial tissue content of Tumstatin. Representative western blots and release patterns of the 75-fragment as strongest band identified by immunoblotting with tumstatin polyclonal antibody.

Tumstatin is the 28 kDa fragment of the NC1-domain of the *α3-chain* of type IV collagen. Immunoblotting showed different bands at 20 kDa and 35 kDa and one major band at 75 kDa (figure 4).

Comparable tissue content for the 75 kDa fragment was found at baseline in all three groups. During mild hypothermic I/R, tissue content of TUM increased without reaching significance at 150 min of reperfusion. In controls, release of the collagen-IV alpha 3-fragment decreased slightly during reperfusion though not statistically significant [control: 1.97±3.05 (Baseline) vs. 1.29±0.22 (30 min REP) vs. 1.31±0.57 (150 min REP; p = 0.22), DeepH: 1.07±0.47 (Baseline) vs. 0.97±0.52 (30 min REP) vs. 1.04±0.58 (150 min REP, p = 0.72), MildH: 1.34±0.17 (Baseline) vs. 1.51±0.39 (30 min REP) vs. 1.75±0.32 (150 min REP; p = 0.07) densitometric units.]

**Figure 5:**
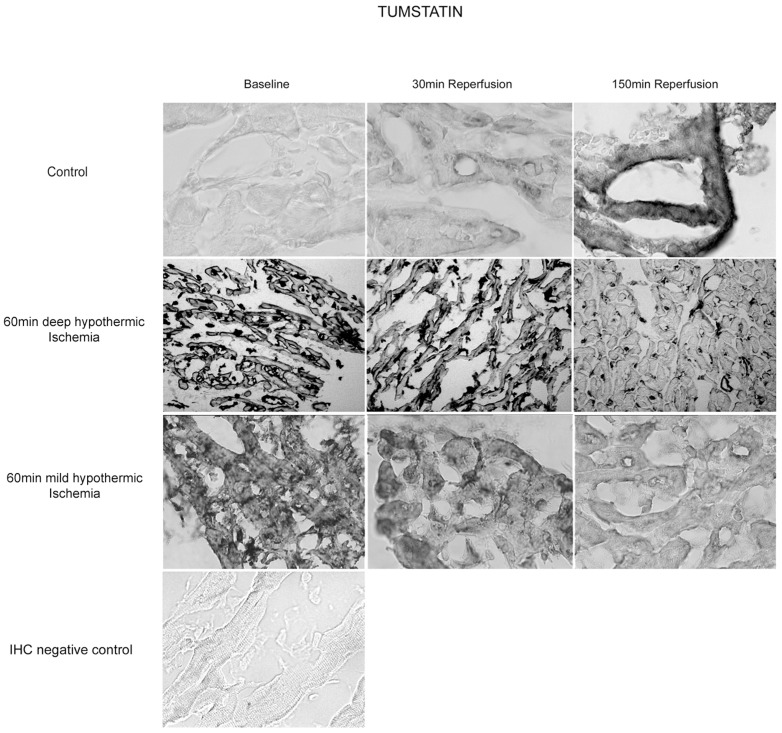
Immunohistochemical staining after deep hypothermic I/R for Tumstatin (Coll4α3) in myocardial sections at baseline, 30 min and 150 min of reperfusion. A significant decrease in antibody binding on the cellular BM is observed at 150

IHC demonstrated a significant decrease of tumstatin antibody binding to >50% of baseline values in both groups during deep hypothermic and mild hypothermic I/R (figure 5, table 2). This observation is in contrast to the time course and localization of ARR antibody binding, thus supporting a differential processing of the collagen-IV-molecule and its α1- and α3- chains depending on external conditions.

#### Tissue content of Endorepellin- Western Blot Analysis and IHC

**Figure 6:**
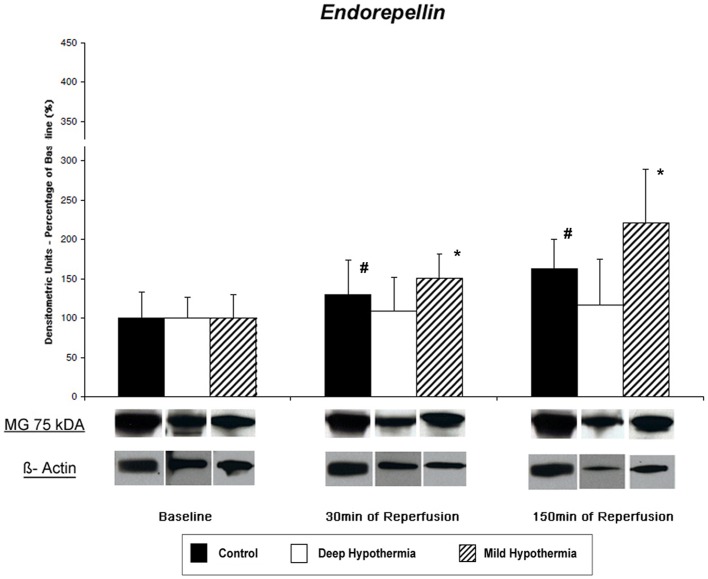
Time couse of endorepellin (LG3) release and representative western blot from degradation Hspg2 during I/R. Non-significant changes of LG3 were observed after deep hypothermic I/R. In contrast, in controls and mild hypothermic I/R a significant upregulation of the 75 kDA-fragment of Hspg2 was observed. ^#^ and * indicate significant intragroup changes (2-way ANOVA).

At baseline a comparable protein content for LG3 was observed in all groups (controls: 0.92±0.31, DeepH: 1.24±0.34, MildH: 1.13±0.34 densitometric units, p = n.s.; Figure 6). After deep hypothermic I/R, a minimal increase of LG3 tissue content was observed, which however was not significant [DeepH: 1.35±0.58 (30 min of REP); 1.45±0.84 (150 min of REP; p = 0.26). In contrast, mild hypothermic I/R resulted in a significant increase of LG3 tissue content of LG3 [1.71±0.53 (30 min of REP); 2.51±1.71 (150 min of REP; p<0.0001). In controls, tissue content of LG3 increased as well [(controls: 1.19±0.52 (30 min REP); 1.50±0.56 (150 min of REP; p = 0.039)].

**Figure 7:**
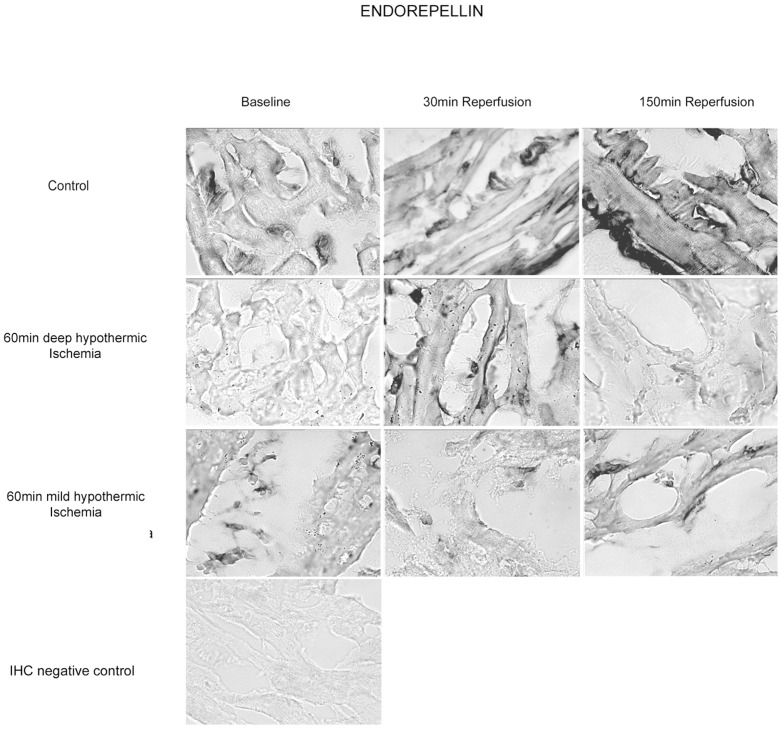
Immunohistochemical staining after deep hypothermic I/R for Endorepellin in myocardial sections at baseline, 30 min and 150 min of reperfusion. A non-significant increase of LG3 staining was observed following deep hypothermic and mild hypothermic I/R, wereas staining in controls remained unchanged.

In contrast to western blot results, IHC demonstrated only minor changes of LG3 polyclonal antibody binding. A non-significant increase of LG3 staining was observed following deep hypothermic and mild hypothermic I/R, wereas staining in controls remained unchanged (Figure 7, Table 2).

#### Immunostaining for Collagen IV – and Hspg2-Epitopes in the Basement Membrane

Intensity of immunostaining for Arresten, Tumstatin and Endorepellin in all groups are detailed in table 2. Figure 6 shows representative cross sections of immunostaining for TUM in DeepH after deep hypothermic I/R at baseline, at 30 min and 150 min of reperfusion. The abluminal endothelial cell side of blood vessels (vascular BM) and the direct surrounding ECM of cardiomyocytes (cellular BM) were histochemical sites of TUM, ARR and LG3 and its precursors. During reperfusion after deep hypothermic ischemia decrease in presentation of TUM at the cardiomyocyte BM were observed. Presentation of ARR and LG3 increased in this group at the cardiomyocyte BM during the first 30 minutes of reperfusion. Following mild hypothermic I/R, ARR, LG3 and TUM similar changes compared to deep hypothermic I/R were observed, however to a lesser extend compared to deep hypothermic I/R. In contrast, presentation of Coll IV and HSPG2 fragments remained unchanged in controls. Preabsorption controls where used to rule out unspecific staining.

## Discussion

Myocardial ischemia and subsequent reperfusion by therapeutic intervention occur frequently in acute coronary syndromes and are strong inductors of myocardial remodelling [5], [30]. Post-ischemic remodelling of the ECM strongly affects patient prognosis as it has been identified as cause of ventricular dilatation with long-term progression to heart failure [13], [29], [32]. After ischemia, a loss of normal ECM architecture by disorganization of matrix deposition and cell-cell contacts has been observed [7], [8], [24]. In this setting, the basement membrane of cardiomyocytes and vascular endothelium plays a key role for matrix reorganization and cell migration and is responsible for structural integrity and mechanical stability of the heart. The present study investigates the effect of acute I/R on processing of BM components resulting in changes in structure and composition of the cardiomyocytes and the vascular endothelium.

Collagen IV and Hspg2 are main structural components of the basement membrane surrounding the vascular endothelium and myocytes in the heart [6], [9]. Both proteins contribute to integrity of blood vessels, prevent leakage of fluids and proteins, and are responsible for mechanical stability and electrical conduction. The ECM fragments ARR and TUM arise from the C-terminal region of collagen IV, whereas LG3 is cleaved from domain V of Hspg2. Their early liberation after ischemia as seen in the present study reflects cleavage of both parental proteins, thus strongly suggesting a functional impact on vascular integrity and mechanical stability. In a recent study in mice, Sasse et al. demonstrated that deficiency of Hspg2 results in extravascular blood leakage and heamopericardium as well as defects during cardiac development [25]. Furthermore, disruption of BM morphology has been demonstrated to impair cell-to-cell communication resulting in disturbance of electrical conductivity with pro-arrythmic effects. Schrickel et al observed, that deficiency of annexin A7, a protein involved in cardiomyocyte membrane structure, results in severe electrical instability in murine hearts [27]. This finding is in line with earlier reports demonstrating impaired electrical conductivity in association with disorganized BM structure [20], [33].

Apart from adverse effects on mechanical stability and electrical conduction, proteolytic fragments liberated from BM constituents are biologically active by inhibiting angiogenesis. The early presence of these fragments following mild hypothermic I/R may potentially suppress vascularization and formation of new vessels in the re-perfused heart, thus contributing to the development of post-ischemic heart failure. For example, endostatin, the 25 kDa-fragment of collagen XVIII, has been characterized in experimental studies and its pro- and anti-angiogenic effects have been reported [21], [26], [34]. Several other matrix-derived degradation products have recently been reported and mediate modulating function in post-ischemic remodelling of the heart. TUM, ARR and LG3 are known as potent inhibitors of angiogenesis. Due to their modulating function, the dynamic changes of release of these fragments are of particular interest during early reperfusion after myocardial ischemia, as this period may offer opportunities for additional therapy in the clinical setting. We recently characterized the *in vivo* post-ischemic release of endostatin by collagen XVIII degradation during early ischemia-reperfusion and demonstrated the highly dynamic changes of release of matrix cleavage in this time period [11]. Endostatin is currently undergoing preclinical trials to investigate its therapeutic potential in tumor therapy [1], [18].

Several new observations were made in the present study. First, we demonstrate an extensive cleavage of BM components during early I/R. Specifically, the acute rise in liberation in ARR and LG3 observed during mild hypothermic I/R attests cleavage of collagen IV and Hspg2 in this group and confirms increased proteolytic activity at this early stage. Together with previous data this supports that ECM cleavage is an early and generalized process during myocardial I/R, affecting several collagen subtypes and non-collagenous proteins of the ECM directly adjacent to the endothelial cells and cardiomoycytes [11]. The data also demonstrate time-dependent differences of release of ARR and LG3 in different temperature conditions. While IHC shows a downregulation of TUM in mild hypothermic and deep hypothermic I/R at the endothelial basement membrane, all other fragments remain unchanged, thus indicating a temperature-dependent processing of the collagen-IV alpha chains and Hspg2 molecule.

Secondly, as demonstrated by immunostaining, I/R is associated with changes in BM structure, a process which seems to be differentially regulated in the BM of cardiomyocytes and endothelial cells and is also impacted by temperature conditions. The differences in IHC-data compared to western blot data further support the hypothesis of differential ECM processing. The presentation of TUM epitopes decreased after mild hypothermic and deep hypothermic I/R, however not in controls. ARR increased after mild hypothermic I/R, however not after deep hypothermic I/R. The differentiated impact of temperature conditions during I/R on BM conformation and protein cleavage may also cause differences on a functional level. While conformational changes of the cardiomyocyte BM may primarily affect mechanical stability and conduction, alterations of the vascular BM would rather effect endothelial function including integrity of blood vessels [5], [15], [25].

The early liberation of matrix cleavage products may be attributed to increased proteolytic activity during early reperfusion. In the myocardium, cleavage of ECM proteins is largely mediated by the matrix-metalloproteinases 2 and 9. Although these enzymes are regulated transcriptionally with stimulation (e.g. through hypoxia) resulting in increased MMP-expression in hours to days, short-term effects are possible and have even been observed *in vivo* in the human myocardium. Spinale at al. demonstrated an increase of MMP proteolytic activity by over 30% from baseline during reperfusion after cardioplegic arrest in patients undergoing elective cardiac surgery [30]. Our finding of increased liberation of matrix fragments early stimulation is also in line with the reports of others. Lalu et al. observed an increase of MMP proteolytic activity in human hearts during coronary artery bypass grafting as early as 10 min of reperfusion. Suhr et al. observed an increased serum level of low-molecular weight matrix fragments immediately after physical exercise, thus confirming the functional regulation of MMPs in skeletal muscle [31].

In further studies it has to be determined whether the release of cleavage fragments from the cardiac BM/ECM is also changed after I/R interventions. This question is of central importance in order to draw a clear picture of ARR, TUM, and LG3 functions. These data would also tell relevant information about the change of vascular permeabilization, which would facilitate the release of cleavage fragments from the BM/ECM into the circulation. It could be speculated that the cardiac-specific release of cleavage fragments under I/R conditions is directly involved in cardiac myocytes, but also in the regulation of peripherally affected tissues.

## Conclusion

The present study provides evidence of differential structural remodelling of the extracellular matrix and the basement membrane in the myocardium after I/R. Mild hypothermic I/R results in a significant liberation of the Col-IV and Hspg2 cleavage products tumstatin, arresten and endorepellin. Mild hypothermic and deep hypothermic I/R both induce conformational changes of the endothelial and cardiomyocyte BM.

These observations demonstrate the differential effect of temperature conditions on cardiomyocyte and endothelial BM structure and cleavage during early I/R. The early structural changes observed in this study may have differentiated functional impact on mechanical stability, electrical conduction and vascular function. Further, the liberation of ARR, TUM and LG3 with biological activity may potentially exert effects during vascularization in the post-ischemic myocardium. As ECM and BM remodelling starts within the first minutes of reperfusion, therapeutic interventions aimed at prevention of post-ischemic heart failure should start early and also focus to preserve BM integrity and structure.

## References

[1] Bendrik C, Karlsson L, Dabrosin C (2010) Increased endostatin generation and decreased angiogenesis via MMP-9 by tamoxifen in hormone dependent ovarian cancer. Cancer Lett 292: 32–40. 19944523 10.1016/j.canlet.2009.11.002

[2] Cailhier JF, Sirois I, Laplante P, Lepage S, Raymond MA, et al (2008) Caspase-3 activation triggers extracellular cathepsin L release and endorepellin proteolysis. J Biol Chem 283: 27220–27229. 18658137 10.1074/jbc.M801164200

[3] Cleutjens JP (1996) The role of matrix metalloproteinases in heart disease. Cardiovasc Res 32: 816–821. 8944811

[4] Creemers EE, Cleutjens JP, Smits JF, Daemen MJ (2001) Matrix metalloproteinase inhibition after myocardial infarction: a new approach to prevent heart failure? Circ Res 89: 201–210. 11485970 10.1161/hh1501.094396

[5] Falk V, Soccal PM, Grunenfelder J, Hoyt G, Walther T, et al (2002) Regulation of matrix metalloproteinases and effect of MMP-inhibition in heart transplant related reperfusion injury. Eur J Cardiothorac Surg 22: 53–58. 12103373 10.1016/s1010-7940(02)00207-5

[6] Hamano Y, Zeisberg M, Sugimoto H, Lively JC, Maeshima Y, et al (2003) Physiological levels of tumstatin, a fragment of collagen IV alpha3 chain, are generated by MMP-9 proteolysis and suppress angiogenesis via alphaV beta3 integrin. Cancer Cell 3: 589–601. 12842087 10.1016/s1535-6108(03)00133-8 PMC2775452

[7] He KL, Dickstein M, Sabbah HN, Yi GH, Gu A, et al (2004) Mechanisms of heart failure with well preserved ejection fraction in dogs following limited coronary microembolization. Cardiovasc Res 64: 72–83. 15364615 10.1016/j.cardiores.2004.06.007

[8] Jugdutt BI (2003) Ventricular remodeling after infarction and the extracellular collagen matrix: when is enough enough? Circulation 108: 1395–1403. 12975244 10.1161/01.CIR.0000085658.98621.49

[9] Kruegel J, Miosge N (2010) Basement membrane components are key players in specialized extracellular matrices. Cell Mol Life Sci 67: 2879–2895. 20428923 10.1007/s00018-010-0367-x PMC2921489

[10] Laplante P, Raymond MA, Labelle A, Abe J, Iozzo RV, et al (2006) Perlecan proteolysis induces an alpha2beta1 integrin- and Src family kinase-dependent anti-apoptotic pathway in fibroblasts in the absence of focal adhesion kinase activation. J Biol Chem 281: 30383–30392. 16882656 10.1074/jbc.M606412200

[11] Lauten A, Ferrari M, Petri A, Ensminger SM, Gummert JF, et al (2009) Experimental evaluation of the JenaClip transcatheter aortic valve. Catheter Cardiovasc Interv 74: 514–519. 19434747 10.1002/ccd.22093

[12] Lauten A, Majos E, Muhlich A, Wahlers T, Weider S, et al (2009) Ischemia-reperfusion injury activates early extracellular matrix processing and expression of endostatin in the heart with differential effects of temperature. Basic Res Cardiol 104: 559–569. 19255800 10.1007/s00395-009-0013-7

[13] Lerman RH, Apstein CS, Kagan HM, Osmers EL, Chichester CO, et al (1983) Myocardial healing and repair after experimental infarction in the rabbit. Circ Res 53: 378–388. 6136345 10.1161/01.res.53.3.378

[14] Maeshima Y, Colorado PC, Kalluri R (2000) Two RGD-independent alpha vbeta 3 integrin binding sites on tumstatin regulate distinct anti-tumor properties. J Biol Chem 275: 23745–23750. 10837460 10.1074/jbc.C000186200

[15] Malan D, Reppel M, Dobrowolski R, Roell W, Smyth N, et al (2009) Lack of laminin gamma1 in embryonic stem cell-derived cardiomyocytes causes inhomogeneous electrical spreading despite intact differentiation and function. Stem Cells 27: 88–99. 18927478 10.1634/stemcells.2008-0335

[16] Maragoudakis ME, Haralabopoulos GC, Tsopanoglou NE, Pipili-Synetos E (1995) Validation of collagenous protein synthesis as an index for angiogenesis with the use of morphological methods. Microvasc Res 50: 215–222. 8538501 10.1006/mvre.1995.1054

[17] Maragoudakis ME, Missirlis E, Karakiulakis GD, Sarmonica M, Bastakis M, et al (1993) Basement membrane biosynthesis as a target for developing inhibitors of angiogenesis with anti-tumor properties. Kidney Int 43: 147–150. 7679456 10.1038/ki.1993.24

[18] Mellon MJ, Bae KH, Steding CE, Jimenez JA, Kao C, et al (2008) Suppression of renal cell carcinoma growth and metastasis with sustained antiangiogenic gene therapy. Hum Gene Ther 19: 487–495. 18507514 10.1089/hum.2007.135 PMC2733371

[19] Mongiat M, Sweeney SM, San Antonio JD, Fu J, Iozzo RV (2003) Endorepellin, a novel inhibitor of angiogenesis derived from the C terminus of perlecan. J Biol Chem 278: 4238–4249. 12435733 10.1074/jbc.M210445200

[20] Morley GE, Vaidya D, Samie FH, Lo C, Delmar M, et al (1999) Characterization of conduction in the ventricles of normal and heterozygous Cx43 knockout mice using optical mapping. J Cardiovasc Electrophysiol 10: 1361–1375. 10515561 10.1111/j.1540-8167.1999.tb00192.x

[21] O'Reilly MS, Boehm T, Shing Y, Fukai N, Vasios G, et al (1997) Endostatin: an endogenous inhibitor of angiogenesis and tumor growth. Cell 88: 277–285. 9008168 10.1016/s0092-8674(00)81848-6

[22] Raymond MA, Desormeaux A, Laplante P, Vigneault N, Filep JG, et al (2004) Apoptosis of endothelial cells triggers a caspase-dependent anti-apoptotic paracrine loop active on VSMC. Faseb J 18: 705–707. 14977881 10.1096/fj.03-0573fje

[23] Roell W, Lu ZJ, Bloch W, Siedner S, Tiemann K, et al (2002) Cellular cardiomyoplasty improves survival after myocardial injury. Circulation 105: 2435–2441. 12021233 10.1161/01.cir.0000016063.66513.bb

[24] Sabbah HN, Sharov VG, Gupta RC, Mishra S, Rastogi S, et al (2003) Reversal of chronic molecular and cellular abnormalities due to heart failure by passive mechanical ventricular containment. Circ Res 93: 1095–1101. 14563716 10.1161/01.RES.0000101932.70443.FE

[25] Sasse P, Malan D, Fleischmann M, Roell W, Gustafsson E, et al (2008) Perlecan is critical for heart stability. Cardiovasc Res 80: 435–444. 18694874 10.1093/cvr/cvn225

[26] Schmidt A, Wenzel D, Ferring I, Kazemi S, Sasaki T, et al (2004) Influence of endostatin on embryonic vasculo- and angiogenesis. Dev Dyn 230: 468–480. 15188432 10.1002/dvdy.20072

[27] Schrickel JW, Brixius K, Herr C, Clemen CS, Sasse P, et al (2007) Enhanced heterogeneity of myocardial conduction and severe cardiac electrical instability in annexin A7-deficient mice. Cardiovasc Res 76: 257–268. 17662970 10.1016/j.cardiores.2007.07.001

[28] Shamhart PE, Meszaros JG (2010) Non-fibrillar collagens: key mediators of post-infarction cardiac remodeling? J Mol Cell Cardiol 48: 530–537. 19573533 10.1016/j.yjmcc.2009.06.017

[29] Spinale FG (2007) Myocardial matrix remodeling and the matrix metalloproteinases: influence on cardiac form and function. Physiol Rev 87: 1285–1342. 17928585 10.1152/physrev.00012.2007

[30] Spinale FG, Koval CN, Deschamps AM, Stroud RE, Ikonomidis JS (2008) Dynamic changes in matrix metalloprotienase activity within the human myocardial interstitium during myocardial arrest and reperfusion. Circulation 118: S16–23. 18824748 10.1161/CIRCULATIONAHA.108.786640 PMC2663795

[31] Suhr F, Rosenwick C, Vassiliadis A, Bloch W, Brixius K (2009) Regulation of extracellular matrix compounds involved in angiogenic processes in short- and long-track elite runners. Scand J Med Sci Sports 10.1111/j.1600-0838.2009.00960.x 19558382

[32] Takahashi S, Barry AC, Factor SM (1990) Collagen degradation in ischaemic rat hearts. Biochem J 265: 233–241. 2154182 10.1042/bj2650233 PMC1136635

[33] van Rijen HV, Eckardt D, Degen J, Theis M, Ott T, et al (2004) Slow conduction and enhanced anisotropy increase the propensity for ventricular tachyarrhythmias in adult mice with induced deletion of connexin43. Circulation 109: 1048–1055. 14967725 10.1161/01.CIR.0000117402.70689.75

[34] Wenzel D, Schmidt A, Reimann K, Hescheler J, Pfitzer G, et al (2006) Endostatin, the proteolytic fragment of collagen XVIII, induces vasorelaxation. Circ Res 98: 1203–1211. 16574906 10.1161/01.RES.0000219899.93384.ed

